# Characterization of an acetyl xylan esterase from the marine bacterium *Ochrovirga pacifica* and its synergism with xylanase on beechwood xylan

**DOI:** 10.1186/s12934-019-1169-y

**Published:** 2019-07-08

**Authors:** Sachithra Amarin Hettiarachchi, Young-Kyung Kwon, Youngdeuk Lee, Eunyoung Jo, Tae-Yang Eom, Yoon-Hyeok Kang, Do-Hyung Kang, Mahanama De Zoysa, Svini Dileepa Marasinghe, Chulhong Oh

**Affiliations:** 1 Korea Institute of Ocean Science & Technology, 2670, Iljudong-ro, Gujwa-eup, Jeju Republic of Korea; 2 Department of Ocean Science, University of Science and Technology, 217 Gajeong-ro, Yuseong-gu, Daejeon, Republic of Korea; 3 Department of Fisheries and Aquaculture, Faculty of Fisheries and Marine Sciences & Technology, University of Ruhuna, Matara, Sri Lanka; 4 College of Veterinary Medicine, Chungnam National University, 99 Daehak-ro, Yuseong-gu, Daejeon, Republic of Korea

**Keywords:** Acetyl xylan esterase, Marine bacteria, *Ochrovirga pacifica*, Synergism, Beech wood xylan

## Abstract

**Background:**

Acetyl xylan esterase plays an important role in the complete enzymatic hydrolysis of lignocellulosic materials. It hydrolyzes the ester linkages of acetic acid in xylan and supports and enhances the activity of xylanase. This study was conducted to identify and overexpress the acetyl xylan esterase (AXE) gene revealed by the genomic sequencing of the marine bacterium *Ochrovirga pacifica*.

**Results:**

The AXE gene has an 864-bp open reading frame that encodes 287 aa and consists of an AXE domain from aa 60 to 274. Gene was cloned to pET-16b vector and expressed the recombinant AXE (rAXE) in *Escherichia coli* BL21 (DE3). The predicted molecular mass was 31.75 kDa. The maximum specific activity (40.08 U/mg) was recorded at the optimal temperature and pH which were 50 °C and pH 8.0, respectively. The thermal stability assay showed that AXE maintains its residual activity almost constantly throughout and after incubation at 45 °C for 120 min. The synergism of AXE with xylanase on beechwood xylan, increased the relative activity 1.41-fold.

**Conclusion:**

Resulted higher relative activity of rAXE with commercially available xylanase on beechwood xylan showed its potential for the use of rAXE in industrial purposes as a de-esterification enzyme to hydrolyze xylan and hemicellulose-like complex substrates.

**Electronic supplementary material:**

The online version of this article (10.1186/s12934-019-1169-y) contains supplementary material, which is available to authorized users.

## Introduction

Hemicellulose is the second most abundant polysaccharide type in land plant cell walls and it consisted of about 25–35% of forest and agricultural residues [1, 2]. Hemicellulose differs from cellulose in its heterogeneous chemical composition associated with β-1,4-xylan, which has high polymerization ability and is highly branched [3]. Hemicellulose consists of a linear backbone of β-1,4-linked xyloses and short-chain branches of *O*-acetyl, α-l-arabinofuranosyl, and α-d-glucuronyl residues [4]. Enzymatic hydrolysis of xylan is catalyzed by endoxylanase and various side chain-cleaving enzymes, such as β-xylosidase, α-glucuronidase, α-arabinofuranosidase, and acetyl xylan esterase [5]. Hardwood xylans (e.g., beechwood and birchwood xylan) are highly acetylated, and acetyl xylan esterase plays a major role in making them partially soluble in water [6] by turning them to short chains through enzymatic degradation. Determining different degradation strategies for these complex and economically important substances is important for their industrial exploitation.

The first use of xylanases [7] obtained from microbes by the pulp and paper industry garnered much interest; this was followed by many studies in this domain over the past few decades [8–10]. Enzymes derived from bacteria [11, 12], including actinomycetes [13, 14] as well as yeast [15, 16], have been used in these studies and are recently being applied at industrial scales [17]. Acetyl xylan esterase facilitates the action of endoxylanases by increasing the approachability to the xylan backbone by cleaving the ester bonds of acetyl groups [6]. Therefore, the synergistic action of acetyl xylan esterase and endoxylanases increases the efficient hydrolysis of xylan [18, 19]. The marine bacterium *Ochrovirga pacifica* belongs to the family Flavobacteriaceae and was identified from a seaweed sample during our previous study [20]. During genome sequence analysis of *O. pacifica*, an acetyl xylan esterase (AXE) gene was found. This study was conducted to characterize this AXE gene and perform cloning, expression, and biochemical characterization of the expressed recombinant enzyme. Finally, the synergistic effect of recombinant AXE was tested with commercially available xylanase on beechwood xylan as the substrate.

## Methods

### Bacterial strains, culture conditions, plasmid, and reagents

*Escherichia coli* DH5α and BL21 (DE3) strains were used as the cloning and expression hosts, respectively. Both strains were grown in Luria–Bertani broth (LB broth) at 37 °C with agitation at 180 rpm. The pET-16b (Novagen, Madison, USA) vector was used for enzyme expression and purification. Buffers and enzymes used for the polymerase chain reaction (PCR) and DNA manipulation were purchased from Takara (Takara Bio Inc., Shiga, Japan). PCR products were purified using an AccuPrep^®^ Gel Purification Kit (Bioneer, Daejeon, South Korea), and cloned plasmids were extracted using an AccuPrep^®^ Plasmid MiniPrep DNA Extraction Kit (Bioneer). Substrates and other reagents used for the enzyme assay, including *p*-nitrophenyl acetate (*p*-NPA), *p*-nitrophenol (*p*-NP), d-xylose, and dinitrosalicylic acid, were purchased from Sigma-Aldrich (St Louis, MO, USA). The synergistic effect was verified with commercially available endo-1,4-β-xylanase derived from *Aspergillus niger* (Megazyme Int., Wicklow, Ireland) using beechwood xylan (Tokyo Chemical Industry Co. Ltd., Tokyo, Japan) as the substrate.

### Identification and molecular characterization of AXE

The marine bacterium *O. pacifica* was isolated from a seaweed sample collected from Chuuk State, Federated States of Micronesia [20], and its genome was sequenced [21]. The AXE gene was identified, and conserved domains were predicted using the National Center for Biotechnology Information (NCBI) Conserved Domain Database (CDD; http://www.ncbi.nlm.nih.gov/cdd/). The Signal IP 4.1 server (http://www.cbs.dtu.dk/services/SignalP/) [22] was used to predict the N-terminal signal peptide of the AXE amino acid sequence, and the EMBOSS Pairwise Sequence Alignment Tool (https://www.ebi.ac.uk/Tools/psa/) [23] was used to calculate the identity, similarity, and gap percentages of the AXE amino acid sequence against the closest neighbor proteins identified by the NCBI BLAST program (https://blast.ncbi.nlm.nih.gov) as well as several acetyl xylan esterases characterized by other works [24]. The isoelectric point and molecular weight were determined using the protein isoelectric point calculator (http://isoelectric.org/calculate.php) [25] and DNA Dynamo (Blue Tractor Software, North Wales, UK), respectively. The nucleotide and amino acid sequence of AXE were submitted to Genbank under accession number MH937751.

### Cloning of the AXE gene

PCR was performed to amplify the targeted AXE gene from the genomic DNA of *O. pacifica* without the predicted N-terminal signal sequence. Forward (GAGAGACATATGCAAAAAGAAGTAAAGTTGGCC) and reverse (GAGAGAGGATCCTTATTCTACTTTGCTTATAGGAAC) primers were designed to bind the pET-16b cloning site using the pET-16b sequence. The PCR mixture consisted of 1 μL of genomic DNA template (200 ng/μL), 35.5 μL sterile deionized water, 5 μL 10× Ex *Taq* buffer (20 mM Mg^2+^), forward and reverse primers (20 pmol each), 4 μL dNTPs (2.5 mM), and Ex *Taq* DNA polymerase (3 U). PCR amplification conditions were as follows: initial denaturation at 94 °C for 5 min; 30 cycles of denaturation at 94 °C for 30 s, annealing at 48 °C for 30 s, extension at 72 °C for 1 min 20 s; and a final extension at 72 °C for 5 min. PCR products were purified using an AccuPrep Gel Purification Kit. The pET-16b vector and purified PCR products were digested with *Nde*I and *Bam*HI restriction enzymes (Takara Bio Inc.) according to the manufacturer’s instructions. Digested PCR product was ligated into the digested pET-16b vector using T4 DNA ligase according to the manufacturer’s protocol. Recombinant plasmid was transformed into *E. coli* DH5α cells by heat shock [26]. Clones of recombinant plasmid were purified using the plasmid extraction kit following the manufacturer’s instructions; it was then transformed to the expression host *E. coli* BL21 (DE3) by heat shock [26]. The nucleotide sequence of the constructed recombinant plasmid was confirmed by sequencing (Macrogen, Korea).

### Protein expression and purification

The clone harboring pET16b-AXE was incubated overnight at 37 °C in 4 mL LB broth supplemented with 100 µg/mL ampicillin (LB-amp); 200 mL LB-amp broth was inoculated with total volume of the overnight culture and incubated at 37 °C with agitation until the optical density at 600 nm reached ~ 0.6. Then, isopropyl-β-d-thiogalactopyranoside (IPTG) was added to a 1 mM final concentration. The culture was incubated for another 24 h at 20 °C under conditions designed to induce expression of the recombinant acetyl xylan esterase (rAXE). Cells were harvested by centrifugation at 8000×*g* for 10 min at 4 °C. Preparation of cell lysate and purification of histidine-tagged rAXE were performed according to the user protocol for the His·Bind^®^ Resin Chromatography Kit (Novagen). Purified protein was quantified by Bradford reagent (Sigma, USA) with bovine serum albumin (BSA) as a standard. The molecular mass and purity were evaluated using 12% Sodium dodecyl sulfate poly-acrylamide gel electrophoresis (SDS-PAGE). A pre-stained protein marker (Lonza ProSieve™, Rockland, USA) was used as the reference.

### Enzyme assay

*p*-NPA was used as the substrate for the rAXE activity assay. The reaction was performed on a microplate at 30 °C in a 200 µL total reaction volume containing 195 µL phosphate buffer (10 mM, pH 6.7), 5 µL purified rAXE enzyme, and 100 µL substrate (0.3423 mM). *p*-NP released within 10 min was measured by monitoring the absorbance at 405 nm (BioTek Instruments, Winooski, USA). *p*-NP was used as the standard. One unit of enzyme activity was defined as the amount of rAXE required to release 1 µmol *p*-NP in 10 min under standard conditions. Each and every assay was performed with a blank contained inactivated enzyme which was run under same pH and temperature conditions.

### Biochemical characterization of rAXE

The optimum temperature and pH were determined using *p*-NPA as the substrate at different temperatures (25–55 °C) and pHs (phosphate citrate buffer for pH 2.0–7.0 and glycine–NaOH buffer for pH 8.0–10.0 at 50 °C) under standard conditions.

### Effect of temperature and pH on rAXE stability

To analyze the temperature stability of rAXE, 5 µL purified enzyme was pre-incubated in 195 µL phosphate buffer (pH 6.7) at 45 °C, 50 °C, and 55 °C over various time durations (0–120 min at 20 min intervals); this was followed by cooling on ice for 5 min. Finally, residual activity was measured under standard assay conditions. To measure the effect of pH on rAXE stability, the enzyme was incubated in a series of buffers (phosphate–citrate buffer for pH 2–7.0 and glycine–NaOH buffer for pH 8–10) at 4 °C for 12 h, and residual activity was tested under standard assay conditions. rAXE without pretreatment was used as the control for both tests.

### Effect of metal ions and salt on rAXE

The effect of different metal ions on rAXE activity was evaluated using 1 mM and 5 mM solutions of Ca^2+^, Mg^2+^, Fe^2+^, Mn^2+^, Cu^2+^, Zn^2+^ and finally ethylenediaminetetraacetic acid (EDTA). The metal ions were incorporated into the buffer solution, and the enzyme activity assay was performed under standard conditions. The relative enzyme activity was calculated using the activity of the untreated sample as 100%. The effect of salt on rAXE was analyzed under standard assay conditions with different NaCl concentrations (0.05–0.5 M). The effect of NaCl on rAXE stability was analyzed by incubating the enzyme (5 µL) in different concentrations of NaCl (0.05–0.5 M) solutions at 4 °C for 12 h and assaying the residual activity of the enzyme in each solution.

### Synergistic effect of rAXE

The synergistic effect of rAXE on the activity of a commercially available endo-1,4-β-xylanase was assayed by measuring the xylose released using a modified 3,5-dinitrosalicylic acid (DNS) method [27] with d-xylose as the standard. The reaction mixture was prepared in an Eppendorf tube containing 1% beechwood xylan in phosphate buffer (pH 8.0), 5 U xylanase, and 1.7 U AXE. Incubation was carried out at 50 °C, and activity was checked at 30 min intervals for 2 h. The amount of enzyme required to produce 1 μmol of reduced sugar per minute was defined as one unit of enzyme activity.

## Results

### Identification and molecular characterization of rAXE

**Fig. 1:**
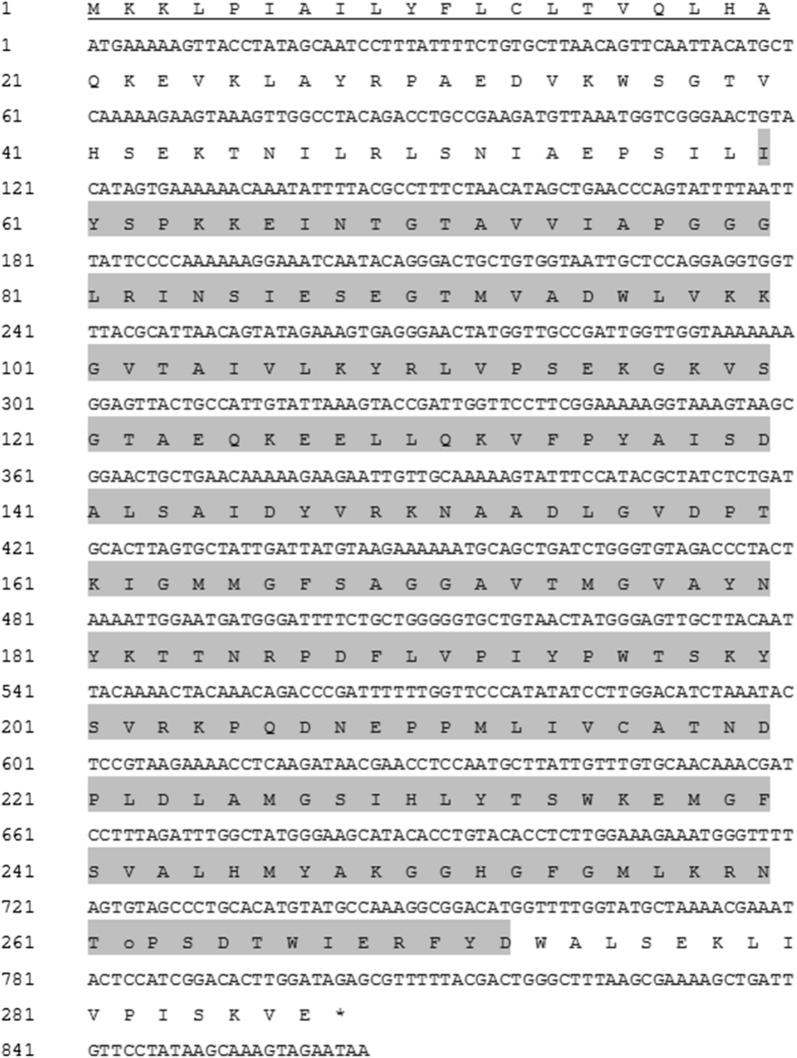
Nucleotide and deduced amino acid sequence of AXE. The N-terminal signal sequence is underlined; the acetyl esterase domain (aa 60 to 274) is highlighted; asterisk indicates a stop codon

The AXE gene has an 864-bp open reading frame that encodes 287 aa the acetyl esterase domain can be found from aa 60 to 274. The predicted molecular mass and isoelectric point were 31.75 kDa and 8.35, respectively. The N-terminal region of the protein contains a signal peptide consisting of 21 aa (Fig. 1). Conserved domain analysis [28] revealed that the enzyme belongs to the alpha/beta hydrolase family and has a conserved acetyl esterase/lipase (COG0657) belonging to the acetyl esterase superfamily. Other nonspecific hits, such as alpha/beta hydrolase fold (pfam07859), dienelactone hydrolase (COG0412), predicted esterase (COG0400), and carboxylesterase type B (COG2272), have also been associated with the sequence. AXE showed the highest sequence identity (55.9%) with an alpha/beta hydrolase of *Wenyingzhuangia fucanilytica* (GenBank Accession No. WP_068826614.1); additional closest matching proteins were 1,4-beta-xylanase (54.9%) of *W. fucanilytica* (ANW96467.1), alpha/beta hydrolase (51.5%) of *Muricauda antarctica* (WP_072877402.1), and alpha/beta hydrolase (51.2%) of *Reichenbachiella versicolor* (WP_109831252.1). These sequences were also compared to amino acid sequences of four characterized acetyl xylan esterases (Table 1).

**Table 1:** Identity and similarity comparisons of the AXE amino acid sequence with its uncharacterized closest neighbor enzymes identified by NCBI BLAST, as well as characterized acetyl xylan esterases from *Bacillus pumilus* [29], *Flavobacterium johnsoniae* UW101 [30], *Butyrivibrio proteoclasticus* B316 [31], *Ruminococcus flavefaciens* 17 [32], and *Streptomyces lividans* 1326 [33]

Organism	Accession no.	Identity (%)	Similarity (%)	Gap (%)	Remarks
*W. fucanilytica*	WP_068826614.1	55.9	70.9	6.0	Uncharacterized
*W. fucanilytica*	ANW96467.1	54.9	68.7	10.4	Uncharacterized
*M. antarctica*	WP_072877402.1	51.5	69.4	5.4	Uncharacterized
*R. versicolor*	WP_109831252.1	51.2	66.1	5.4	Uncharacterized
*B. pumilus*	CAB76451.2	14.9	27.7	49.8	Characterized
*F. johnsoniae UW101*	ABQ06890.1	14.1	24.01	43.05	Characterized
*B. proteoclasticus B316*	ADL35669.1	10.1	19.4	63.3	Characterized
*R. flavefaciens 17*	CAB55348.1	8.3	13.3	71.5	Characterized
*S. lividans 1326*	AAC06115.2	7.3	12.7	70.7	Characterized

### Expression and purification of rAXE

**Fig. 2:**
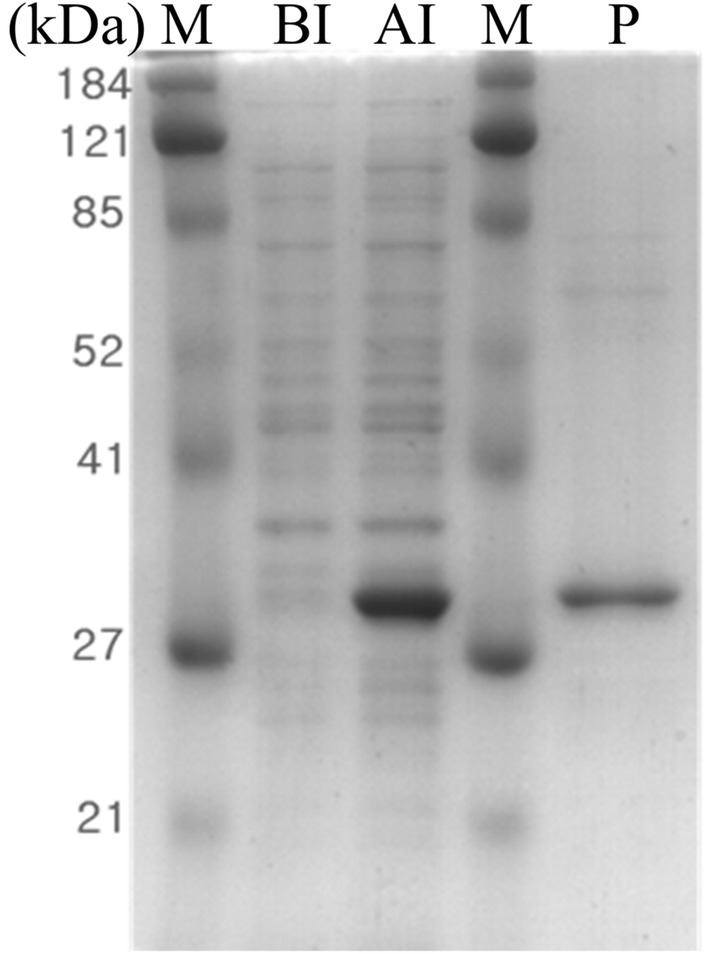
SDS-PAGE analysis of rAXE. *M* molecular weight marker, *BI* whole cell lysate before induction, *AI* whole cell lysate after induction (incubated at 20 °C for 16 h with 180 rpm agitation), *P* purified rAXE using His·Bind^®^ Resin Chromatography Kit

The AXE gene was expressed in pET-16b vector with an N-terminal 10-histidine tag supplied with the vector to facilitate the affinity purification of the target protein. Figure 2 shows the high expression level and purity of the recombinant protein, which gave a strong band on SDS-PAGE. The protein was purified by one-step affinity purification using a His·Bind^®^ Resin Chromatography Kit (Novagen). The molecular mass of the protein estimated by SDS-PAGE was ~ 32.0 kDa, roughly equal to the predicted molecular mass (32.1 kDa).

### Biochemical characterization of rAXE

**Fig. 3:**
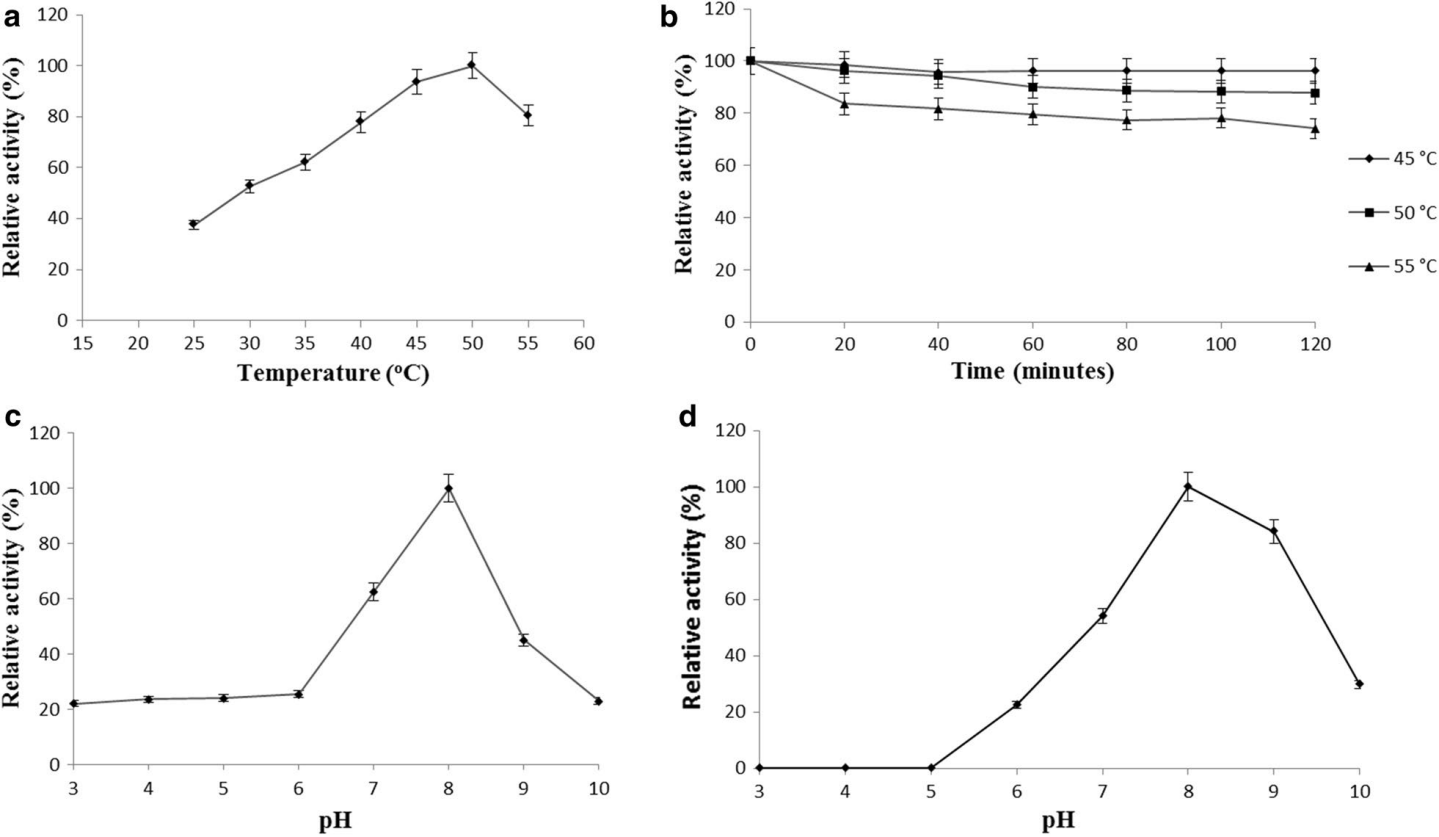
Effects of pH and temperature on rAXE activity. **a** Effect of temperature on relative enzyme activity (relative activity was calculated using activity at 50 °C as 100%). **b** Thermal stability assay (relative activity was calculated using activity of untreated enzyme as 100%). **c** Effect of pH on activity at 50 °C (relative activity was calculated using activity at pH 8.0 as 100%). **d** pH stability assay (relative activity was calculated using activity of enzyme treated with pH 8.0 buffer as 100%). Data are shown as mean ± standard deviation (sd), n = 3

The optimal temperature for rAXE was 50 °C; its activity decreased around 20% at temperatures > 50 °C (Fig. 3a). The thermal stability assay showed that rAXE maintains its residual activity almost constantly throughout and after incubation at 45 °C for 120 min (Fig. 3b). Incubation at 55 °C for 20 min reduced its activity by more than 50%. rAXE showed maximum activity at pH 8.0 (assayed at 50 °C) and exhibited less than 25% relative activity at pHs < 6.0 (Fig. 3c). Activity was drastically decreased above pH 8.0, and 23% relative activity was observed at pH 10.0. After 12 h incubation at 4 °C and pH 3.0–5.0, there was 0% remaining enzyme activity. Residual activities at pHs 7.0, 8.0, and 9.0 were > 40%; stability dramatically decreased at pHs > 9.0 (Fig. 3d). The maximum specific activity of rAXE towards *p*-NPA was recorded at pH 8.0 (assayed at 50 °C) as 40.08 U/mg.

### Effect of metal ions and salt on rAXE activity

**Fig. 4:**
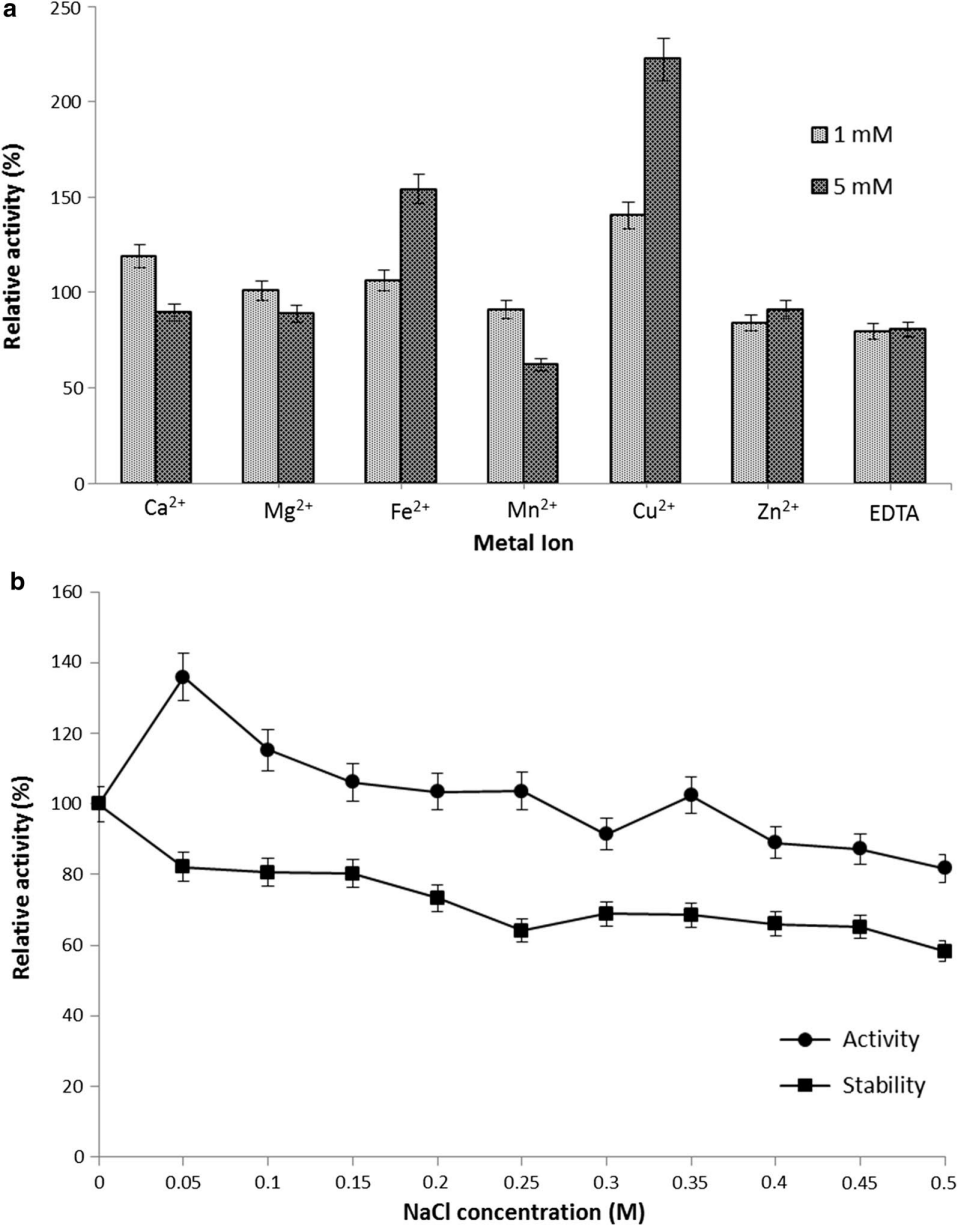
**a** Effect of various metal ions on the relative activity of rAXE. The enzyme reaction was performed with final concentrations of 1 mM and 5 mM for each metal ion. The activity in the absence of a metal ion was taken as the control (100%). **b** Effect of NaCl on the relative activity and stability of rAXE. The activity at 0 M NaCl in the reaction mixture was taken as the control (100%). Data are presented as mean ± standard deviation (sd), n = 3

The effect of metal ions on rAXE activity was determined using the activity of the untreated enzyme as the control (100%). Relative activity was examined at two concentrations (1 mM and 5 mM) of each metal ion. Reaction mixtures containing 1 mM Ca^2+^, 1 or 5 mM Cu^2+^, and 5 mM Fe^2+^ showed strong stimulatory effects on enzyme activity; 5 mM Ca^2+^, 5 mM Mg^2+^, both 1 mM and 5 mM Mn^2+^ or Zn^2+^ and EDTA showed inhibitory effects on enzyme activity (Fig. 4a). The effect of NaCl on rAXE activity and stability was examined; as shown in Fig. 4b, enzymatic activity was strongly stimulated at 0.05 M NaCl, whereas activity gradually decreased at higher concentrations (Fig. 4b). However, relative activity higher than 100% was recorded at NaCl concentrations between 0.05 and 0.25 M. Incubation in NaCl for 12 h had no positive effect on enzyme activity, and the residual activity for all concentrations was < 100% relative activity.

### Synergistic effect of rAXE

**Fig. 5:**
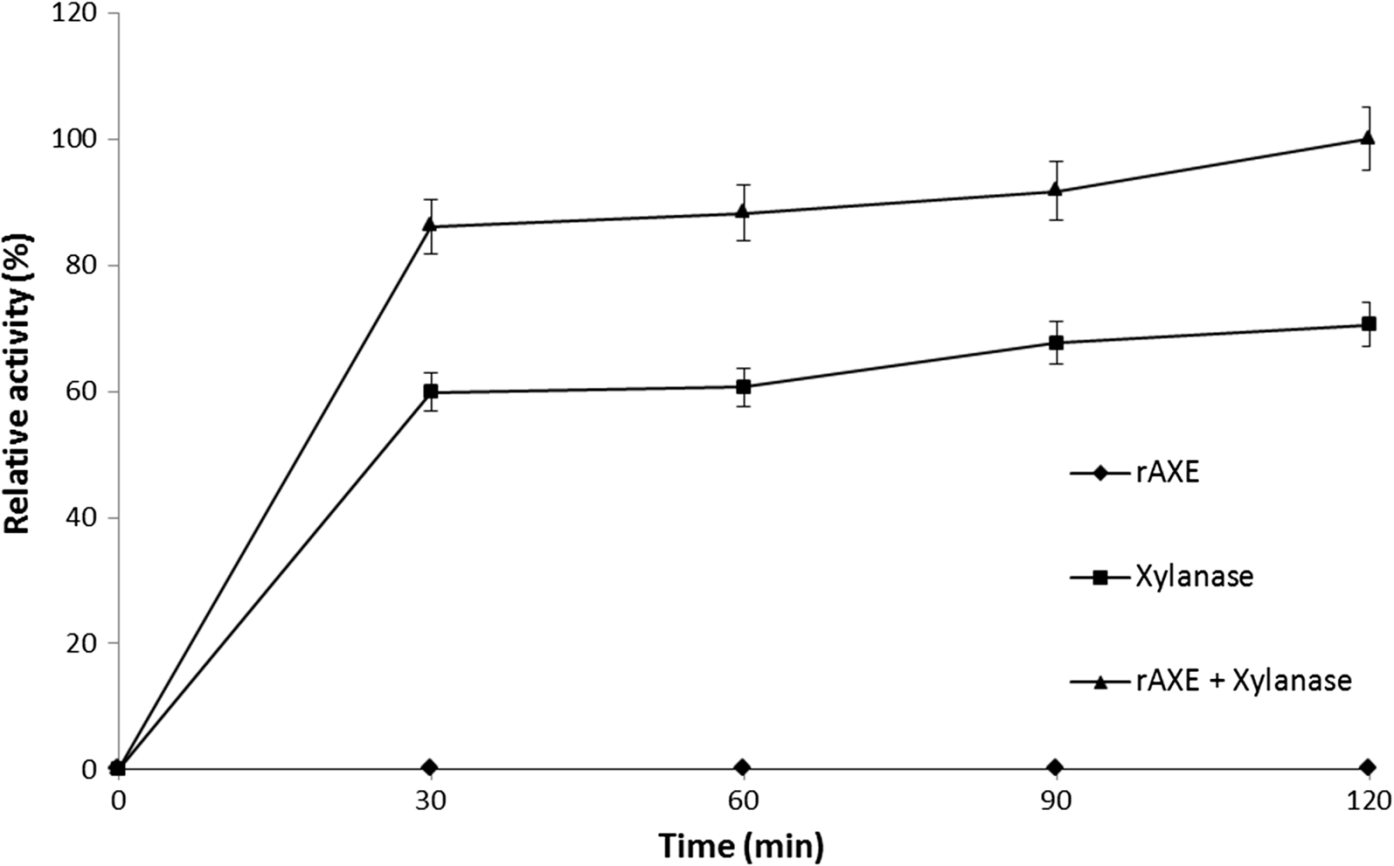
Synergism of AXE with a commercially available xylanase on beechwood xylan as the substrate. The reaction mixtures were prepared in Eppendorf tubes containing 1% beechwood xylan in phosphate buffer (pH 8.0) and incubated over the time at 50 °C. Combination of rAXE and xylanase was included 1.7 U of rAXE and 5 U of commercially available endo-1,4-β-xylanase derived from *A. niger*. Only rAXE and xylanase contained reaction mixtures were included 1.7 U and 5 U respectively. Relative activity was determined with the activity of rAXE + xylanase at 120 min as 100%. Data are given as means ± SD, n = 3

The synergistic effect of rAXE on the activity of a commercially available xylanase was assayed (Fig. 5). The reaction mixture prepared with rAXE only (1.7 U) showed no relative activity; xylanase only (5 U) showed ~ 70% relative activity. The reaction mixture containing both xylanase and rAXE showed 29.47% higher relative activity compared to xylanase alone (a 1.41-fold increase).

## Discussion

This study was conducted to characterize the acetyl xylan esterase gene from the marine bacterium *O. pacifica*, isolated from a seaweed sample [20]. This is the first report of such an enzyme from the genus *Ochrovirga* (family: *Flavobacteriaceae*), and further biochemical characterization of the expressed enzyme within an *E. coli* expression system was performed. Genomic DNA analysis of *O. pacifica* revealed the presence of the AXE gene, and NCBI conserved domain analysis [34] revealed the acetyl esterase domain within the amino acid sequence (aa 60–274). The acetyl esterase domain belongs to the alpha/beta hydrolase superfamily. This superfamily consists of lipases, peroxidases, proteases, epoxide hydrolases, and dehalogenases, which are functionally diversified for hydrolyzing different substrates [35]. Several other nonspecific hits, such as alpha/beta hydrolase protein fold, were also associated with the amino acid sequence, which may have developed evolutionarily to hydrolyze substrates with different chemical and physicochemical properties. The highest sequence identity (55.9%) matched the uncharacterized alpha/beta hydrolase of *W. fucanilytica* (GenBank Accession No. WP_068826614.1), demonstrating the uniqueness of the AXE gene of *O. pacifica*. A phylogenetic tree of the AXE amino acid sequence along with the closest matches identified by BLAST and five characterized acetyl xylan esterases reported previously showed that AXE from *O*. *pacifica* is positioned in a separate clade (Additional file 1: Fig. S1). The family *Flavobacteriaceae* is well known for the degradation of macromolecules, such as complex carbohydrates [36]. Most species of the family grow on algal thalli and have the ability to degrade algal cells [37]. Razeq et al. [30] have characterized and reported an acetyl xylan esterase encoded in the *Flavobacterium johnsoniae* genome (FjoAcXE), which is a member of same family. However, the phylogenetic relationship between AXE and FjoAcXE is not close (see Additional file 1).

The presence of the N-terminal signal sequence suggests that the AXE enzyme may be secreted for extracellular hydrolysis processes; expression for the experiment was done without the signal sequence. The optimum temperature of rAXE was 50 °C, and it maintained more than 80% of its residual activity after incubation for 2 h at 50 °C. In this experiment optimum temperature was examined within 25 °C to 55 °C range. Reason was substrate converted to products rapidly in higher temperatures. Previously characterized acetyl xylan esterases from different microorganisms [19, 38] showed optimal temperatures < 50 °C and lower thermal stability compared to rAXE; this may be advantageous in industrial applications of rAXE. The optimal pH of rAXE at the optimal temperature was pH 8.0. Most fungal and rumen bacterial-derived acetyl xylan esterases show neutral optimal pHs [39, 40], whereas bacteria living in harsh conditions and marine environments show alkaline pHs of 8.0–9.5 [29, 41, 42]. Characteristics of the enzymes and proteins derived from the microbes are highly depended on their ecological conditions [43–45]. Naturally they use their enzymes to degrade the substrates in environment and play a major role in nutrient cycling [46]. At present there is an increasing attention on the marine microbial enzymes because of their good performances in hard conditions like high or low temperatures, pressure, pH and high salt concentrations [47, 48].

The effect of different metal ions on the activity of rAXE was evaluated. Strong stimulation was observed with the presence of 1 mM of Ca^2+^, 1 and 5 mM Cu^2+^, and 5 mM Fe^2+^ in the reaction mixture. Further studies of metal ion interactions with rAXE are recommended. According to Taylor et al. [49], some acetyl xylan esterases from microorganisms show special metal ion preferences that enhance their catalytic activities. Furthermore, 5 mM Ca^2+^, 5 mM Mg^2+^, and both 1 and 5 mM Mn^2+^ and Zn^2+^ showed inhibitory effects on enzyme activity; the inhibitory effect of Zn^2+^ on esterase has been reported by several authors [50, 51]. The effects of salt on rAXE activity and stability were tested and 0.05 M NaCl strongly stimulated rAXE activity. Most esterases derived from microorganisms are more salt tolerant than rAXE; enhanced activity has been demonstrated within 0.0–4.0 M NaCl [52, 53]. According to Steiner and Lindskog [54], the stimulatory activity of salt occurs due to the increased chemical potential of *p*-NPA in aqueous solutions rather than participation in the enzyme-related catalytic steps.

The synergistic effect of xylanase and rAXE on beechwood xylan was investigated. The cooperative effect of rAXE and xylanase on xylan initiates with the de-esterification of acetyl substitutes on side chains, making them more accessible to xylanase for hydrolysis of the glycosidic bonds [2]. Shorter polysaccharide fragments resulted from the increase in the substrate preference of rAXE attributable to xylanase activity [6]. Furthermore, decreased polymerization caused by xylanase may increase the reaction rate by decreasing the viscosity of the reaction mixture, allowing more substrate-rAXE interactions [18]. In our study, the mixture of rAXE and xylanase exhibited a 1.41-fold increase in relative activity on beechwood xylan compared to xylanase alone. Therefore, rAXE has good potential as an accessory enzyme for hydrolyzing xylan and hemicellulose.

## Conclusion

The present study identified and biochemically characterized rAXE from the marine bacterium *O. pacifica*, which was isolated from a seaweed sample. This is the first report of such an enzyme from the genus *Ochrovirga*. The synergistic activity of rAXE with commercially available xylanase showed higher relative activity on beechwood xylan. Therefore, there is strong potential for the use of rAXE in industrial purposes as a de-esterification enzyme to hydrolyze xylan and hemicellulose-like complex substrates.

## Additional file

**Additional file 1.** Phylogenetic analysis of *O*. *pacifica* AXE along with the uncharacterized closest amino acid sequences identified by NCBI BLAST and several characterized acetyl xylan esterases. The neighbor-joining tree was constructed using the bootstrap method with 1000 replications.

## Data Availability

All data generated or analyzed during this study are included in this published article and its Additional file.

## Abbreviations

AXE: acetyl xylan esterase
rAXE: recombinant acetyl xylan esterase
*p*-NPA: *p*-nitrophenyl acetate
*p*-NP: *p*-nitrophenol
SDS-PAGE: sodium dodecyl sulfate-polyacrylamide gel electrophoresis
BSA: bovine serum albumin
DNS: 3,5-dinitrosalicylic acid
EDTA: ethylenediaminetetraacetic acid
FjoAcXE: acetyl xylan esterase encoded in the *Flavobacterium johnsoniae* genome
