# Supplementary material for: Characterization of an acetyl xylan esterase from the marine bacterium Ochrovirga pacifica and its synergism with xylanase on beechwood xylan
Source: Microb Cell Fact. 2019 Jul 8;18:122. doi: 10.1186/s12934-019-1169-y (PMC6615230; doi:10.1186/s12934-019-1169-y)
Supplement: Supplementary file 1 — Additional file 1. Phylogenetic analysis of O. pacifica AXE along with the uncharacterized closest amino acid sequences identified by NCBI BLAST and several characterized acetyl xylan esterases. The neighbor-joining tree was constructed using the bootstrap method with 1000 replications. [file 12934_2019_1169_MOESM1_ESM.docx]

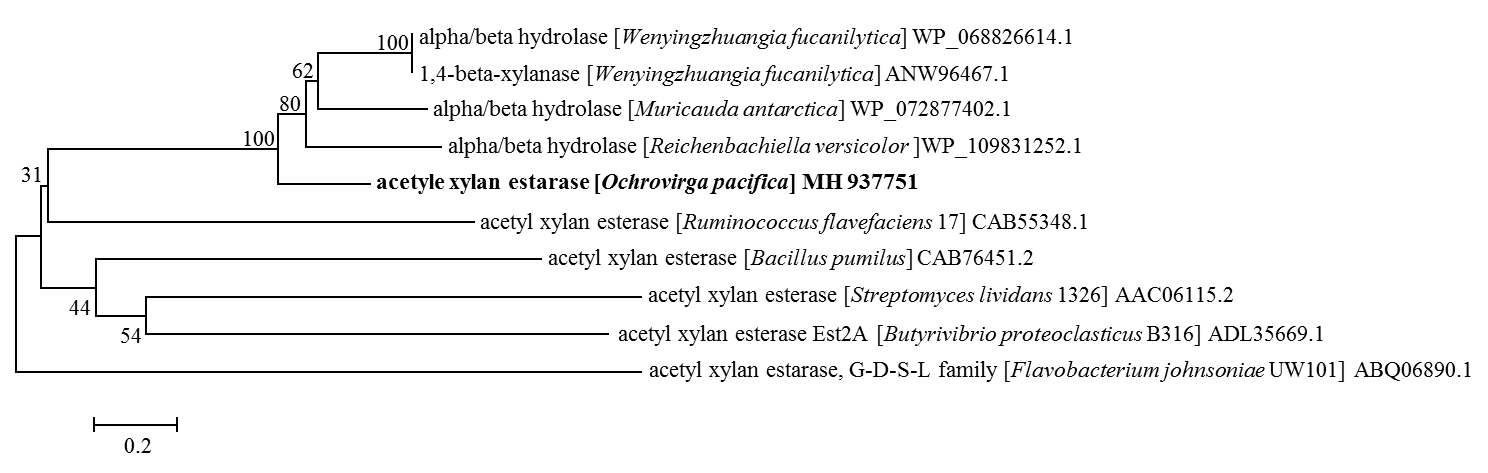


**Additional file 1.**  Phylogenetic analysis of *O*. *pacifica* AXE along with the uncharacterized closest amino acid sequences identified by NCBI BLAST and several characterized acetyl xylan esterases. The neighbor-joining tree was constructed using the bootstrap method with 1000 replications.
